# Collision tumor in the thyroid gland, "even with" Hashimoto's thyroiditis: a double-edged sword in thyroidology?

**DOI:** 10.1590/1806-9282.20240146

**Published:** 2025-03-17

**Authors:** Ilkay Cinar, Ilker Sengul

**Affiliations:** 1 Giresun University, Faculty of Medicine, Department of Pathology – Giresun, Turkey.; 2 Giresun University, Faculty of Medicine, Division of Endocrine Surgery – Giresun, Turkey.; 3 Giresun University, Faculty of Medicine, Department of General Surgery – Giresun, Turkey.

Dear Editor,

Thyroid malignancies, per se, are the most prevalent type of endocrine malignancy, and papillary thyroid carcinoma (PTC) is reported as the seventh most common cancer in women. It disperses through lymphatic spread within the gland and occurs in 20% of multifocal and contralateral carcinomas^1–7^. The incidence of Hürthle cell carcinoma (HCC), first portrayed by Ewing in 1928, is less than 5% of all thyroid carcinomas. Hürthle cells are oncocytes characterized by mitochondria-rich, vesicular nuclei, prominent nucleoli, and large acidophilic granules in the cytoplasm; most are benign adenomas, while up to 40% are malignant. HCC is diagnosed with capsular/vascular invasion or lymphatic spread, similar to follicular carcinoma, and is more aggressive and resistant to radiodine compared to other differentiated thyroid carcinomas^8–10^.

Hashimoto's thyroiditis (HThy) is the most prevailing begetter of hypothyroidism and thyroid autoimmune disease in iodine-deficient areas worldwide, in which gradual destruction of the gland due to lymphocytic infiltration and apoptotic processes resulting from the inflammatory response caused by the development of autoantibodies arise. Nevertheless, thyroid carcinomas may develop on the background of HThy and are often associated with PTC. Continuous and persistent inflammatory reactions may damage cells by forming reactive oxygen species (ROS). Furthermore, mutations leading to DNA damage caused by ROS outcome in thyroid carcinomas. Moreover, BRAF mutation and rearranged during transfection (RET)/PTC rearrangement have been identified in PTC, while RET/PTC rearrangement has been demonstrated in other tumors, including HCC and non-carcinomatous diseases like HThy. However, the liaison between HThy and thyroid carcinoma, particularly PTC, remains controversial to date^9–12^. Of note, PTC and HCC can occur separately on the background of HThy, but their coexistence is extremely rare. In this case, we would like to mention a 45-year-old female with neck sonography revealing a heterogeneous isoechoic nodule filling the entire left lobe, 36×27 mm, and two heterogeneous nodules in the right, the largest 10×15 mm. Her fine-needle aspiration of the nodule in the left lobe expressed moderate cellularity involving entirely Hürthle cells with atypia, revealing Hürthle cell type follicular neoplasm (Figure 1). Furthermore, she underwent a total thyroidectomy, and her macroscopic examination revealed a widespread appearance consistent with HThy in the glandular structure, along with a cream-colored lesion of 15 mm in the right lobe, and a hemorrhagic cystic nodule of 35 mm in the left. Moreover, her histopathological evaluation revealed (i) the presence of HThy, (ii) a conventional variant of PTC in a 15-mm nodule in the right lobe (Figure 2), and (iii) HCC in a 35-mm nodule in the left lobe (Figures 3 and 4). Herein, we faced an extreme case of a collision tumor, including PTC and HCC, even though both phenomena developed on the background of HThy.

A collision tumor is the simultaneous presence of two or more histologically different tumors in the same organ. Various hypotheses have been proposed regarding their development. Some suggestive hypotheses are as follows: (i) the "incidental colocalization" theory, different tumors can develop side by side with the same effect; (ii) the first tumor leads to the development of the second tumor by altering the microenvironment; and (iii) different tumors originate from a typical stem cell. Collision tumors can be observed in the thyroid gland and many other organs, such as the lung, stomach, and breast. Thyroid neoplasms usually involve a single type, and thyroid collision tumors are less frequent than others, affecting less than 1% of all. To date, combinations of PTC, medullary thyroid carcinoma, follicular thyroid carcinoma, follicular adenoma, and squamous cell carcinoma have been reported^13–19^.

A posteriori, HCC is more aggressive and resistant to radioiodine ablation than PTC. Thus, the treatment, like their diagnoses, of collision tumors is also complicated due to their multifaceted pathology and biological behavior. Therefore, personalized management of the therapeutic approach can be applied as each component is evaluated separately. Collision tumors remain a noteworthy and frightening issue in thyroidology in case of any skimpiness in vigilance for the providers of thyroid health.
